# The American Medical Association

**Published:** 1881-05

**Authors:** 


THE AMERICAN MEDICAL ASSOCIATION Did a handsome thing before adjourning at Richmond, in electing Dr. Joseph J. Woodward, U. S. Army, President of that body for the present year.

Dr. W. is not only an able writer and microscopist, but in every respect, one of the ablest and most accomplished members of the profession. The honor was all the greater, as he was not in attendance upon the meeting, and probably had no idea of his name being mentioned in connection with the position. We are glad to learn from the reports of the proceedings, that the abler members of the profession favor the revival of the lancet in the treatment of pneumonia and other inflammatory affections. A decade will not have past until January, 1882, since we had the temerity, as some of our best friends thought, to advocate, in unequivocal language, a revival of this long neglected but most potent and valuable remedial agent, in an article published in the *Medical and Surgical Reporter* of Philadelphia. And now, notwithstanding the potential influence of fashion and prejudice against its reintroduction into practice, we have the great satisfaction of knowing that almost all the able thinkers and practitioners in the medical profession in this country and Europe, are either practicing or instructing others on the important and, often indispensable utility of sanguineous evacuation in inflammation.

We take great pleasure and satisfaction in referring our readers to our paper on blood-letting, as published in the *Reporter*, in January, 1872, and republished in *this* journal last year, page 476. A *small* faction may require a decade or more before they become convinced of the utility of blood-letting, but we feel assured that all intelligent medical practitioners at the present day will readily endorse what we said ten years ago, viz.: "That the necessity for the use of the lancet is as great at the present time as it ever was in the past, and that the type of disease has undergone no such change as to render the abstraction of blood unnecessary or improper in the successful management of all cases attended with a full, tense and quick pulse." In fact, we are more fully satisfied, if possible, in regard to the facts and arguments set forth in that brief paper, now, than ever before, and though no prophet, nor the son of a prophet, we confidently predict for blood-letting with the lancet, and other means, a higher place in our remedial agents, within the next decade than it ever held before within the history of our therapeutical resources. It will not be used so indiscriminately as in the past, but more scientifically and successfully than ever before in the history of the healing art.

The officers of the association for the current year are as follows, viz.:

President, Dr. J. J. Woodward, U. S. A.

Vice-Presidents, Doctors P. O. Harper, Arkansas; L. Conner, Michigan; Eugene Gressom, North Carolina, and Hunter McGuire, Virginia.

Secretary, Dr. Wm. B. Atkinson, Pennsylvania.

Treasurer, Dr. R. J. Dunlison, Pennsylvania.

Librarian, Dr. Wm. Lee, Washington.

To fill vacancies in the Medical Council, Doctors S. N. Benham, Pennsylvania; J. M. Toner, Washington; D. A. Linthecum, Arkansas; William Brodie, Michigan; H. D. Holton, Vermont; A. B. Sloan, Missouri, and R. B. Cole, California.

St. Paul, Minnesota, was selected as the place for the next Annual Meeting; and Dr. Ston was appointed chairman of the Local Committee of Arrangements.

Dr. Billings, U. S. A., moved the adoption of the report of the committee, paid a high compliment to Dr. Woodward, the nominee for President, and thanked the committee, in behalf of the Medical Staff of the Army, for the compliment paid it in the selection of one of its most honored members for the highest position in the gift of the Association.

